# Kidneys for Sale: Empirical Evidence From Iran

**DOI:** 10.3389/ti.2022.10178

**Published:** 2022-06-24

**Authors:** Tannaz Moeindarbari, Mehdi Feizi

**Affiliations:** Department of Economics, Faculty of Economics and Administrative Sciences, Ferdowsi University of Mashhad, Mashhad, Iran

**Keywords:** live kidney transplantation, kidney market, organ sales, compensated donation, Iranian model of kidney transplantation

## Abstract

The kidney market in Iran is the only legal market of this sort globally. Yet, it has not been empirically studied based on real data. For the first time, we obtained data on donors and recipients from the Kidney Foundation in Mashhad, April 2011 up to March 2018, and assessed which individualistic characteristics contribute to a kidney’s price. Our findings indicate that each year of education for both donors and recipients increases the kidney price. Moreover, old patients are willing to make a higher payment to young vendors. We have also provided some policy implications to improve the efficiency of kidney allocations.

## Graphical Abstract

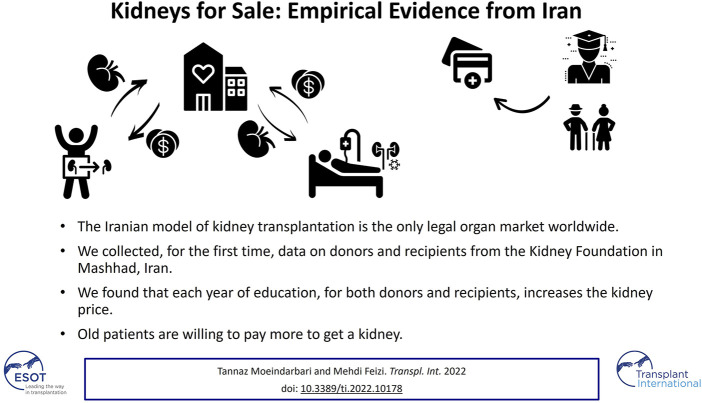

## Introduction

The insufficient philanthropic supply of organs has led to a significant organ shortage, mounting transplant waiting lists, and many renal patients losing their lives throughout the world. None of the new approaches to increasing the kidney donor pool in developed countries, such as developing deceased donation, introducing kidney exchange programs, and optimizing the allocation algorithms, have been successful in eliminating the drastic shortage of transplantable kidneys. Nevertheless, market-based arrangements to increase donations of human organs are broadly considered unacceptable from ethical perspectives and are therefore not relevant in almost all countries (1).

Since kidney markets are illegal everywhere, except Iran, there is very little known about the consequences of such a market. This paper studies the monetary market for kidneys in Mashhad, the second-largest kidney market in Iran, after the market in Tehran. Our analysis is based on a unique inclusive dataset of this market for about 7 years. For the very first time to the best of our knowledge, we assess which individualistic characteristics and institutional factors could explain a realized price of a kidney. We shed light on its several socio-economic aspects and provide evidence that gives readers a better understanding of how a monetary market for organs could work and its pros and cons.

A kidney market can considerably release patients from suffering under dialysis, increase their lifetimes, and cut healthcare costs. Nevertheless, such a market creates some ethical concerns, and our analysis should not be seen as an authorization for it. Many opponents of a market for kidneys are concerned that the two sides of the market are divided by wealth, where the majority of buyers are the rich, and most sellers are the poor who sell their kidneys because they desperately and sometimes urgently need money. That is why some opponents argue that a market for organs can be coercive (2).

However, we should note that as a kidney market had not made potential donors poor, it should not be blamed for that. Such a market provides a costly signal, i.e., selling one’s kidney, that make desperate poor people visible. Therefore, a kidney market could even provide a truthful mechanism to distinguish poor people and do something for them. After all, we do not expect that a wealthy individual sells his/her kidney just to get financial support from the government.

Nevertheless, a market for organs can have a crowding-out effect on intrinsic motivations for an altruistic kidney donation. Our data from the Kidney Foundation, KF hereafter, in Mashhad confirm this concern as very few kidneys have been donated altruistically. The KF is a non-profit, volunteer-run charitable organization that mediates between recipients and donors to assist both and further applies for related government and charitable benefits with no incentives for making the pairs.

Another concern about the kidney market is that low-income patients might not be able to afford live kidneys. However, as the KF in Iran is a charity in the first place, it subsides poor patients to get a kidney. Moreover, we could design a market where the government is the only authority that could legally purchase kidneys and then allocate them similar to how cadaver kidneys are allocated. Our collective responsibilities for people who suffer from kidney failure are best accomplished through a government-monopsony market in kidneys where the government is the only buyer who distributes kidneys based on need, but not ability to pay (3). In this way, we treat all patients equally, and they all have equal access to kidneys, disrespectful of their wealth level.

Notably, this is a self-financing scheme since savings from dismissing patients from dialysis and shrinking the waiting list for kidneys are much more than the costs of purchasing live kidneys. Spending even a portion of this saving on improving the living conditions of donors, e.g., post-transplant medical care, and special social services, reduces the long-term adverse effects of kidney transplantation for donors while saves many lives without irreparable damage to others.

The paper is organized as follows. In Section *Related Literature*, reviews the related literature. Section *The Iranian Experience: The Case of Mashhad* explains in short how the Iranian model of the kidney market works. Section *Data Analysis* introduces data and analyzes it descriptively, reports and discusses multivariate regressions, and provided some policy implications. Section *Conclusion* concludes.

## Related Literature

Several U.S. states have legislated laws providing leave or tax benefits to organ and bone marrow donors and their employers. The passage of tax incentive legislation increased living unrelated kidney donation rates in New York (4). However, this legislation works for moderately invasive procedures such as bone marrow donation, but it cannot increase the quantity of organ donation, which is more hazardous and troublesome (5).

Organ sales ban forces the organ trade underground, strengthens the role of organ brokers, and lessens organ sellers’ bargaining power, leaving them exposed to even higher levels of exploitation (6). The urgent monetary destitution for the poor, who commonly do not have appropriate access to the financial market, gives them no other choice than vending their organ. In this regard, it seems impossible to stop the illegal organ trade. Regulating the market minimizes harm by making it possible to scrutinize the market, to enforce compliance with standards that protect both donors and recipients, and to remove greedy dealers, thus enabling the poor to receive transplants on an equal footing with the rich (7).

Regulated and incentivized systems that eliminate impediments to donation and remunerate donors could raise donations and reduce the unregulated markets and their harms. Working Group on Incentives for Living Donation suggest standards and guidelines for such a donation mechanism that would do more good than harm. Its critical components are protection, regulation, oversight, and transparency under the auspices of the appropriate government or government-recognized body (8).

There are some concerns about the long-term well-being of kidney donors. They are at increased risk of long-term risk for end-stage renal disease, ESRD hereafter, cardiovascular, and all-cause mortality compared with a control group of non-donors who were eligible for donation (9, 10). Therefore, prospective donors must be fully and adequately informed about the consequences of a kidney transplant (11).

The US public is potentially amenable to compensating kidney donors (12). They supports limited incentives for living donation while ethnic minorities and low-income Whites are more accepting of specific monetary incentives. Most of them favored reimbursement of medical costs, paid leave, and priority on the waiting list for living donation (13). Most of the ESRD patients are willing to pay for a kidney while male, ailing and wealthy patients are more willing to pay (14).

However, not all renal patients are willing to accept an altruistic live-donor transplant since they do not perceive an opportunity for direct reciprocity. Some feel either unworthy of an altruistic live-donor transplant or responsible for the risks to an altruistic donor. Therefore, receipt of an altruistic transplant might be an even more complicated decision than a donation (15). Since altruism is significantly related to donor motivation only for donations to direct family members, limited material incentives may be necessary for improving donations among individuals unrelated to kidney transplant recipients (16).

Some studies proposed a monetary incentive for living donors that would increase organs supply, discharge waiting in massive queues, raise the quality of life, and put an end to thousands of needless deaths (17–19). They estimated that a price of $15,000 per living donor would be enough to eliminate the shortage of kidneys and the waiting list in the US. Even paying a more substantial figure of $45,000 for living donors and $10,000 for deceased donors has far more benefits than costs (20), since $5,000 and $10,000 are the Median lowest monetary compensation that would urge to donate for relatives and strangers, respectively, while with ten times more money, one could no longer decline to donate (21). Based on donors’ data from the most extensive online kidney matching point in Iran, and naturally around the globe, most kidney donors are male, around 31 years old, having an average willingness to accept of almost 12,400 USD (22).

Based on individual-level data from the United States and the European Union collected in 2001–2002, individuals who were familiar with the organ donation process or even had just some encounter with the health system were more likely to become organ donors, while minorities were less likely to donate (23). Mother’s education also had a significant positive effect on organ donation. The decision to be an organ donor is affected by relational ties, religious beliefs, cultural influences, family controls, body integrity, knowledge about the organ donation process, and previous interactions with the health care system, e.g., medical mistrust, and fear of early organ retrieval (24).

## The Iranian Experience: The Case of Mashhad

The Iranian model of kidney transplantation, IMKT hereafter, established in 1988, is an example of a compensated and regulated living unrelated renal donation. It is an efficient and ethical model that can be employed by all other countries, which currently lack the necessary regulatory supervision (25). The IMKT has provided a unique opportunity for socio-economic analysis of a market for organs, which has not been fairly addressed.

In line with the Declaration of Istanbul, DoI hereafter, organ trafficking and transplant tourism are prohibited in the IMKT. It authorizes monetary compensation for kidney transplantation but does not tolerate transplant commercialism. Commercialism refers to the possibility within the free-market system to abuse vulnerable people to make a private profit. However, donors in the IMKT are not exploited, but they are supported by law and protected by medical insurance. Therefore, the IMKT adheres to the DoI.

Since April 2000, when the Iranian parliament passed the Organ Transplantation and Brain Death Act that approved deceased organ donations, the share of transplants from deceased donors has firmly risen to more than half of transplants. Nevertheless, there are other legal barriers, e.g., the consent of all close related families for the transplantation right after the death, making the deceased organ donations not enough to eliminate the excess demand for kidneys. Even with a supply of live kidneys from the monetary market, patients in Iran should still wait for months to receive a kidney for transplantation.

The IMKT includes a compensation negotiated directly between the recipient and living donor. In Iran, the word that is used for kidney vendors is donor, though they get paid. We use the same tradition in this paper but have in mind the tautology. Additionally, the government pays a reward to donors, a fixed 10 million Rials, equal to about 1,200 USD at that time and 150 USD at present, called the gift of altruism. Every few years, the Kidney Foundation of Iran announces a new official floor price for a kidney that each of 39 branches of the KF in each province is obligated to follow. This fixed price is independent of individualistic features such as gender and health status. However, the government has allowed an additional payment above this threshold negotiated directly between the patient and living donor.

The legal kidney market in Iran is not working the same in all cities. On the one extreme, it has its remarkable function in Mashhad with transparent side payments (26). In Mashhad, the KF tries to prevent the poor from unadvisedly selling their kidneys by informing them about the consequences of a kidney transplant, fixing their financial needs, and imposing several legal obstacles before a transplant is authorized (27). These measures exclude a majority of potential donors who want to sell their kidneys and address the concern that the poor might sell their kidneys without explicitly knowing the health consequences of their decision. On the other extreme in Shiraz, the prohibition of payment beyond the official national rate has naturally fostered a black market for kidneys. Donors and recipients in such a market surreptitiously exchange money under the table while they had signed an agreement assuring that no payment would be made over the official rate.

Any ESRD patient with no willing related donors is referred by a physician’s letter to the corresponding KF in that province where s/he could enter the kidney waiting list. Each potential kidney donor also registers at the KF after undergoing the preliminary medical tests and bringing the notarized consent of him/herself and his/her family. There are four different matching lines for each blood type, and a donor is paired with the first renal patient in the same blood type line, based on the first-come/first-served, who is matched in terms of Human Leukocyte Antigens.

Although this matching mechanism is not the most efficient one, it raises the chance of a successful transplant. Nevertheless, this is not the only way of matching, and both sides could publicly advertise and find each other outside the KF. However, since nephrologists discourage patients from contacting random donors and transplantation centers only accept donors referred by KF, both donors and recipients have to register there and go through the required paperwork and medical tests.

Once any matched pair agrees on a price, payment is made through the KF by sending a letter to the transplantation centers located at university hospitals under the scrutiny of the *Ministry of Health and Medical Education*. The government also pays for all transplant-related expenses and provides donors with medical coverage for 1 year after the nephrectomy and even military service exemption in case it applies. Therefore, in contrast to other organ markets in developing countries, the medical team has no share of the money paid by the recipient to the donor (28). Nevertheless, the recipient bears the main payment burden as the governmental compensation has remained fixed since its initiation in 1998 and is now worth about one-eighth.

As a result of the Iranian system of compensated donation, the number of renal transplants conducted has substantively enhanced such that from about a decade afterward, the renal transplant waiting list has been almost eliminated, (29) and most of the Iranian kidney transplant candidates, irrespective of their socioeconomic class, have access to kidney transplantation (28). The Iranian system, despite its success, has definite defects and shortcomings, such as stigmatization of donors, (30) which deter donors from following up their medical status, crowding out effect which defeats altruistic and prosocial donation, (31, 32) commercialization and commodification, (33) which exploits the poor and disrespect human integrity (34).

## Data Analysis

We collected 436 paired kidney donors and recipients from April 2011 (the beginning of the year 1,390 in Persian Calendar) up to March 2018 (the end of the year 1,396 in Persian Calendar) KF in Mashhad, the second most populated city in Iran. In Mashhad, the realized side payment to donors beyond the official floor price is exchanged through the KF and documented in both donors’ and recipients’ profile. This procedure makes the kidney market in Mashhad unique, while in other major markets in main cities of Iran such as Tehran, Shiraz, and Kermanshah, there is no such data. Table 1 shows the descriptive statistics of our data. The average kidney price is about 134.5 million Rials (almost 4,400 USD), significantly higher than the average floor price, about 97.9 million Rials (almost 3200 USD), and less than 2 years of work with the minimum level of wage (35).

**TABLE 1:** Descriptive statistics.

Variables	Mean	S. D	Min	Max
**Price (million Rials)**	134.52	57.29	50	450
**Donor Age**	29.91	4.78	20	40
**Patient Age**	37.94	13.46	8	68
**Donor Years of Education**	8.04	3.71	0	16
**Patient Years of Education**	9.09	5.07	0	22

A large number of the available studies suggest that most donors are female, while the majority of recipients are male (36–39). Women might perceive organ donation as their motherly responsibility or spousal obligation to save their suffering child or partner. (35) They may be more likely to demonstrate altruistic nurturing behavior, (37, 41) more vulnerable to be influenced by family pressure to donate, and less able to resist this burden (37, 40, 42).

However, kidney vending may secure low-status women in the Middle East from being forced to serve as altruistic family donors (43). The kidney market in Iran is biased and favors women because they are less likely to donate and more likely to receive a kidney. As men are traditionally supposed to be the breadwinner of the family in Iran, they have prevalence among donors. There are more male and married donors in our dataset than recipients (almost 85% male and 79% married in donors, and about 65% male and 74% married in recipients). Donors were also mostly literate, with 8 years of education at secondary school, on average (35).

Donors tend to be poor young married men, who are financially motivated towards donation, but recipients are unfortunately not that wealthy, as 47% of them were unemployed. Interestingly, we had five closely related donors who sold their kidneys, albeit at much lower prices. We made a dummy variable for these cases. These descriptive statistics confirm the similar picture illustrated already in the literature that showed between 84% and 90% of living unrelated renal donors were male, 80% were married, and the majority were at the level of high school education (44, 45).

We found various education levels, e.g., primary, secondary, high school, and Bachelor, for both donors and recipients. In Iran, the education system used to have 5 years of primary school, 3 years of secondary school, 3 years of high school, and 1-year of pre-college. However, we realized in our data that having any education level does not necessarily mean that one has indeed finished that level. Instead, he or she was mostly about to get to that level. We considered the average years of education at each level for those who claimed they educated up to that level. Namely, we considered three, seven, and 10 years of education for primary, secondary, high school levels of education.

We adjusted the kidney price with the Iranian Central bank’s monthly consumer price index to make data from different years comparable in a pooled setting and takes its logarithm as the dependent variable. Our regressions in Table 2 illustrate that each extra year of education for both donors and recipients, as a proxy for their income level, raises the kidney price, although the intensity of increase varies. Each extra year of education for a donor compared to a patient has double effects on the kidney price and increases it by 0.8 million Rials (almost 26.2 USD).

**TABLE 2:** OLS regressions on the logarithm of inflation-adjusted kidney price.

Variables	Model I	Model II	Model III	Model IV
Constant	4.19*** (0.025)	4.089*** (0.038)	4.074*** (0.039)	4.078*** (0.039)
2012	0.024 (0.035)	0.023 (0.035)	0.03 (0.035)	0.034 (0.035)
2013	−0.045 (0.038)	−0.045 (0.038)	−0.044 (0.038)	−0.04 (0.038)
2014	0.077** (0.036)	0.075** (0.036)	0.077** (0.036)	0.084** (0.036)
2015	0.084** (0.035)	0.077** (0.034)	0.081** (0.034)	0.085** (0.034)
2016	0.095** (0.039)	0.101*** (0.038)	0.103*** (0.038)	0.103*** (0.038)
2017	0.117** (0.045)	0.099** (0.045)	0.1** (0.045)	0.1** (0.044)
Donor Education		0.007*** (0.002)	0.008*** (0.002)	0.008*** (0.002)
Recipient Education		0.004** (0.002)	0.004** (0.002)	0.004* (0.002)
Age Difference			0.001** (0.000)	0.001** (0.000)
Relative Donor				−0.265*** (0.096)
N	432	432	431	431
R_squared	0.052	0.079	0.087	0.104
Adjusted R-squared	0.039	0.062	0.068	0.082
Prob (F-statistic)	0.000	0.000	0.000	0.000

However, as it is distinct from Table 1, donors tend to be relatively less educated than recipients (on average about 1 year, with no degree higher than Bachelor). Therefore, each extra year of additional education has a higher level of marginal effect on their income, especially given that they are relatively more impoverished. This difference in the effect of education on price might also reflect the difference between patients’ willingness to pay and donors’ willingness to accept. After all, donors should be much more averse to losing their organs than those about to receive ones.

Moreover, donors compared to recipients tend to be relatively younger, about 8 years on average. However, patients have wider variations in their age, as it is not restricted, and after all, the disease could emerge at any age, and it is more probable for elders, while donors’ age has much less variance since it is restricted by law to be between 18 and 40 years old. There are different views on the effect of age on graft survival and, consequently, the kidney’s price. While kidney allocation mechanisms do not consider factors other than blood type and tissue compatibility, the market mechanism itself considers each pair’s age difference. Table 2 indicates that the age difference between donor and recipient in each pair significantly augments the kidney price. Namely, when a kidney from a young donor is assigned to an old patient, the price is significantly higher compared to another case where the old patient gets a kidney from an old donor. A younger donor can receive a larger payment, up to about 100 thousand Rials (almost 3.25 USD), for each year of the age difference.

According to the estimation results, a family relationship between the donor and the patient reduces the kidney price. A related donor, who decides not to donate his or her organ for free, vends it to his or her relative for about 26.5 million Rials (about 867.15 USD) less than non-related donors. The dummy variables of all years, except 2012 and 2013, raise kidney prices in all models. This robust and positive effect could be because, compared to the official price in 2011, in these 2 years, the official prices increased a little, from 60 million Rials (almost 1963.35 USD) to 70 million Rials (almost 2,290.5 USD) and 90 million Rials (almost 2,945 USD) respectively, while afterward, it increases to 140 million Rials (almost 4,581.15 USD).

## Conclusion

A market for organs is a typical example of market failure where the market equilibrium does not maximize social welfare. Iran is the only country in the world where it is not illegal to exchange an organ, e.g., a kidney, for money. The only government intervention so far in Iran’s kidney market has been setting a minimum price for the whole country. While there is a scoring system for patients with renal disease in Iran that prioritize them getting a kidney from a deceased donor, Iran’s kidney market does not prioritize patients and works simply on the first-come-first-serve basis. This paper is the very first attempt to provide a cornerstone to regulate the kidney market more efficiently.

We tried to explain variations in kidney price based on individualistic characteristics such as age and education level. Our findings indicate that related donors, who need to be compensated, vend their kidneys to close relatives for significantly less monetary compensation. We could interpret this impact as the crowding-out effect. Moreover, each year of education for both donors and recipients increases the kidney price. While kidney allocation mechanisms do not consider factors other than blood type and tissue compatibility, the market mechanism itself considers age difference and allows a higher price for assigning a kidney from a young donor to an old patient. These findings call for a revised mechanism for the Iranian kidney market that should not be merely based on the similarity of blood types, but also it is supposed to consider individual characteristics of donors and recipients such as age.

## Data Availability

The datasets presented in this article are not readily available because access to data is restricted to protect proprietary information. It can be made available upon request with permission of the kidney foundation in Mashhad. Requests to access the datasets should be directed to feizi@um.ac.ir.

## Abbreviations

DoI, Declaration of Istanbul; ESRD, End Stage Renal Disease; IMKT, Iranian Model of Kidney Transplantation; KF, Kidney Foundation.
